# Monophosphoryl Lipid a as an adjuvant for immune therapy? A detailed *in vitro* comparison to LPS

**DOI:** 10.1186/2045-7022-4-S2-O21

**Published:** 2014-03-17

**Authors:** Stefan Schülke, Lothar Vogel, Adam Flaczyk, Sonja Wolfheimer, Stefan Vieths, Stefan Scheurer

**Affiliations:** 1 Paul-Ehrlich-Institut, Paul-Ehrlich Straße 51-59, Langen (Hessen), 63225, Germany; 2 Paul-Ehrlich Institut, Division of Allergology, Langen, Germany

## 

Monophosphoryl lipid A (MPL) is a non-toxic TLR4 ligand, derived from Salmonella minnesota R595 (Re) lipopolysaccharide (LPS) by chemical modification. It is clinically used as an adjuvant for cancer treatment (Fendrix^®^, Ceravix^®^) and allergen specific immunotherapy (Pollinex^®^ Quattro, ORALVAC^®^). Nevertheless, reports on the mechanism of adjuvant activity are limited. The aim of this study was to compare the immune modulating capacities of MPL and LPS *in vitro*.

In both human and murine lung epithelial cell lines (LA-4, A549) LPS induced a higher CCL2 secretion than MPL. In murine BM-derived myeloid dendritic cells (mDC), LPS as well as MPL stimulation resulted in the same pattern of cytokine secretion (IL-1β, IL-6, IL-10 and TNF-α). At high concentrations of MPL, IL-1β secretion was 4-fold higher compared to LPS, whereas LPS stimulation resulted in higher secretion of IL-6, IL-10 and TNF-α, respectively. Moreover, mDC stimulation with both adjuvants resulted in a pronounced cell activation pattern characterized by CD40 and CD69 upregulation, at which LPS proved to be more potent than MPL (thresholds for mDC activation: MPL: 100 ng/ml, LPS: 1 ng/ml). In MyD88^-/-^ and Trif^-/-^ mDC, MPL-induced cytokine secretion was absent in MyD88- but only reduced in Trif-deficient mDC. LPS induced cytokine secretion was mostly unchanged in Trif^-/-^ mDC. Furthermore, the co-administration of MPL and Ova resulted in enhanced IFN-γ and IL-5 secretion from OVA-specific DO11.10 CD4^+^ T cells co-cultured with BALB/c mDC which was not observed for LPS controls. In line with this result, stimulation with a covalent fusion protein of MPL and Ova (MPL:Ova) resulted in enhanced cytokine secretion from both mDC (IL-1β, IL-6, TNF, IL-10, IL-12) and CD4 T cells (IL-5, IL-13, IL-2, IFN-γ, IL-17) compared to equimolar concentrations of MPL and Ova provided individually or as a mixture. Interestingly, Ova induced IL-9 secretion from CD4^+^ T cells was dose-dependently repressed when fused to MPL.

In summary, using *in vitro* assay systems we observed similar but attenuated immune responses induced by MPL in comparison to LPS. MPL applied together with allergen (either mixed or covalently fused) on CD4^+^ T cells boosted allergen-specific TH1-, TH2-, and TH17-adaptive responses. Although considered safe in humans, further studies should critically assess the adjuvant capacity of MPL in order to evaluate potential non-desired immunological effects.

